# Out-of-Field Hippocampus from Partial-Body Irradiated Mice Displays Changes in Multi-Omics Profile and Defects in Neurogenesis

**DOI:** 10.3390/ijms22084290

**Published:** 2021-04-20

**Authors:** Simonetta Pazzaglia, Barbara Tanno, Francesca Antonelli, Paola Giardullo, Gabriele Babini, Prabal Subedi, Omid Azimzadeh, Zohaib N. Khan, Kateryna Oleksenko, Fabian Metzger, Christine von Toerne, Damien Traynor, Dinesh Medipally, Aidan D. Meade, Munira Kadhim, Fiona M. Lyng, Soile Tapio, Anna Saran, Mariateresa Mancuso

**Affiliations:** 1 Laboratory of Biomedical Technologies, Agenzia Nazionale per le Nuove Tecnologie, l’Energia e lo Sviluppo Economico Sostenibile (ENEA), 00123 Rome, Italy; barbara.tanno@enea.it (B.T.); francesca.antonelli@enea.it (F.A.); paola.giardullo@enea.it (P.G.); annasaran60@gmail.com (A.S.); 2 Department of Physics, University of Pavia, 27100 Pavia, Italy; gabriele.babini@unipv.it; 3 Department of Woman and Child Health and Public Health, Fondazione Policlinico Universitario A. Gemelli, Istituto di Ricovero e Cura a Carattere Scientifico (IRCCS), 00168 Rome, Italy; 4 Helmholtz Zentrum München, German Research Center for Environmental Health GmbH (HMGU), Institute of Radiation Biology, D-85764 Neuherberg, Germany; psubedi@bfs.de (P.S.); omid.azimzadeh@helmholtz-muenchen.de (O.A.); zohaib.khan@helmholtz-muenchen.de (Z.N.K.); kateryna.oleksenko@helmholtz-muenchen.de (K.O.); soile.tapio@helmholtz-muenchen.de (S.T.); 5 Federal Office for Radiation Protection, Section Radiation Biology, D-85764 Oberschleissheim, Germany; 6 Research Unit Protein Science, HMGU, D-85764 Neuherberg, Germany; fabian.metzger@helmholtz-muenchen.de (F.M.); vontoerne@helmholtz-muenchen.de (C.v.T.); 7 Radiation and Environmental Science Centre, Technological University Dublin, D02 HW71 Dublin, Ireland; damien.traynor@tudublin.ie (D.T.); dinesh.medipally@mytudublin.ie (D.M.); Aidan.meade@tudublin.ie (A.D.M.); fiona.lyng@tudublin.ie (F.M.L.); 8 Department of Biological and Medical Sciences, Oxford Brookes University (OBU), Oxford OX3 0BP, UK; mkadhim@brookes.ac.uk

**Keywords:** MiRNome, proteomics, metabolomics, dentate gyrus, radiation, hippocampal neurogenesis, ionizing radiation

## Abstract

The brain undergoes ionizing radiation exposure in many clinical situations, particularly during radiotherapy for brain tumors. The critical role of the hippocampus in the pathogenesis of radiation-induced neurocognitive dysfunction is well recognized. The goal of this study is to test the potential contribution of non-targeted effects in the detrimental response of the hippocampus to irradiation and to elucidate the mechanisms involved. C57Bl/6 mice were whole body (WBI) or partial body (PBI) irradiated with 0.1 or 2.0 Gy of X-rays or sham irradiated. PBI consisted of the exposure of the lower third of the mouse body, whilst the upper two thirds were shielded. Hippocampi were collected 15 days or 6 months post-irradiation and a multi-omics approach was adopted to assess the molecular changes in non-coding RNAs, proteins and metabolic levels, as well as histological changes in the rate of hippocampal neurogenesis. Notably, at 2.0 Gy the pattern of early molecular and histopathological changes induced in the hippocampus at 15 days following PBI were similar in quality and quantity to the effects induced by WBI, thus providing a proof of principle of the existence of out-of-target radiation response in the hippocampus of conventional mice. We detected major alterations in DAG/IP3 and TGF-β signaling pathways as well as in the expression of proteins involved in the regulation of long-term neuronal synaptic plasticity and synapse organization, coupled with defects in neural stem cells self-renewal in the hippocampal dentate gyrus. However, compared to the persistence of the WBI effects, most of the PBI effects were only transient and tended to decrease at 6 months post-irradiation, indicating important mechanistic difference. On the contrary, at low dose we identified a progressive accumulation of molecular defects that tended to manifest at later post-irradiation times. These data, indicating that both targeted and non-targeted radiation effects might contribute to the pathogenesis of hippocampal radiation-damage, have general implications for human health.

## 1. Introduction

The hippocampus is a highly radiosensitive brain structure involved in forming, organizing and storing memories. Its critical role in the pathogenesis of radiation-induced neurocognitive dysfunction is well recognized. Inhibition of adult hippocampal neurogenesis following whole-brain irradiation is considered one of principal mechanisms of radiation-induced cognitive dysfunction [1,2]. In addition, a negative correlation between radiation dose to the hippocampus and neurocognitive functions in children receiving cranial irradiation has also been demonstrated [3]. Consequently, hippocampus avoidance during whole-brain radiotherapy seems to be promising in helping to preserve the cognitive function [4].

Radiation effects, however, are not confined to directly irradiated tissues, rather living organisms cope with injury through coordinated cell/tissue responses. Over the last 20 years, the classical nuclear target paradigm of radiation biology has been challenged by the emerging role of non-targeted effect of radiation. In fact, irradiation has been shown to affect not only the cells traversed by radiation track, but also non-irradiated neighboring cells, a response described as radiation-induced bystander effects (RIBE). Communication between irradiated and sham-irradiated neighboring cells, involving molecular signals via intercellular communication or through soluble secreted factors produced by irradiated cells, initiates RIBE and out-of-field (abscopal) effects [5]. In vitro and in vivo experimental studies imply cytokines, miRNAs, protein kinases and exosomes as well as oxidized DNA among the clastogenic factors secreted from irradiated cells that in turn affect the expression of genes, proteins and epigenetic regulation in bystander cells [6]. However, in vivo data on out-of-target responses necessary to elucidate physiological cellular connections within a tissue or cross-talk among tissues are still scarce. Determining the contribution of targeted and off-target effects in the clinic is still challenging. This has important consequences not only in radiotherapy but also possibly in diagnostic procedures and in radiation protection.

Our previous work provided examples of in vivo out-of-target oncogenic radiation responses by showing that cancer development in brain of *Ptch1^+/−^* mice, a genetically sensitive mouse model, was increased by radiation exposure of distant tissues, indicating that there is a level of communication between irradiated and non-irradiated tissues and organs [7]. Noteworthy, the decrease of tissue communication by ablation of one copy of *Connexin 43* (*Cx43*) gene, reduced the bystander tumor response in *Ptch1^+/−^* mice [8]. A few in vivo examples of brain radiation-induced bystander non-cancer effects have also been reported in the literature. In a study examining the impact of non-brain directed radiation therapy on the brain, a global brain glucose hypometabolism, as well as acute and persistent multifocal neuroinflammation, were reported in exposed mice [9]. Brain bystander effects after low-dose liver irradiation, manifested as altered gene and protein expression and DNA damage associated with neuroanatomical and behavioral changes, have also been reported in rats [10,11]. Finally, altered brain morphology after focal irradiation of neonatal mice (8 Gy), specifically targeting white matter (anterior commissure), neuronal (olfactory bulbs) or neurogenic (subventricular zone) regions, revealed that radiation damage locally can have important off-target consequences for brain development [12]. However, a global understanding out-of-target brain radiation-induced effects, especially within the context of an intact mammalian organism, has been lacking.

In this study, we aimed at investigating the key mechanisms of out-of-target radiation-induced effects in the hippocampus, through a multiomic approach, by comparing the changes in non-coding RNAs, protein and metabolic levels as well as in the rate of dentate gyrus (DG) neurogenesis induced by targeted and non-targeted irradiation. To this purpose, brains of C57Bl/6 mice were collected 15 days and 6 months after whole body (WBI) or partial body (PBI) irradiation with 0.1 Gy or 2.0 Gy of X-rays. PBI was performed by exposing the lower third of the mouse body, whilst the upper two thirds were shielded. Results showed nearly identical changes in non-coding RNAs, proteins and rate of neurogenesis 15 days after WBI and PBI with 2.0 Gy, indicative of the existence of out-of-target brain radiation responses in vivo. We also investigated the long-term consequences of in-field and out-of-field exposure at 6 months post-irradiation, finding that while most of the WBI effects were permanent or progressive, the majority of PBI-induced changes were only transient or decreasing. Finally, we identified marked differences in the timing of manifestation of hippocampal defects depending on radiation dose. Noteworthy, in vivo investigations on radiation responses in the hippocampus of conventional mice, fully recapitulating the physiological conditions, allowed unravelling of the mechanistic features of targeted and non-targeted radiation responses, providing a greater understanding of RIBE and of its clinical implications in the pathogenesis of radiation-induced hippocampal neurocognitive dysfunction.

## 2. Results

### 2.1. MiRNome Analysis after Direct or Bystander Radiation Exposure of the Hippocampus

**Figure 1:**
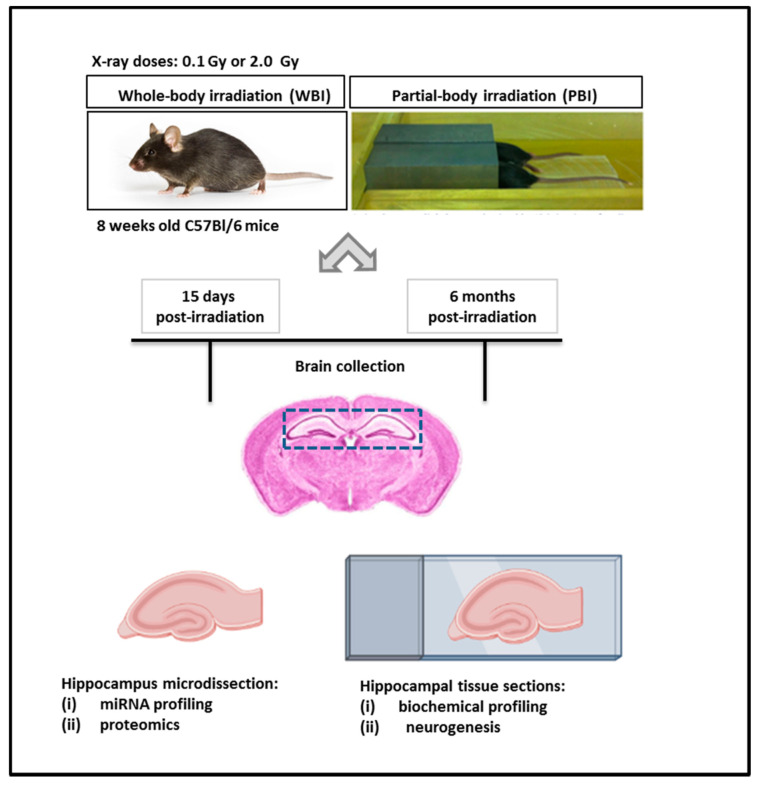
Exposure system and experimental design scheme. Here, 8 week-old C57Bl/6 female mice were subjected to WBI or PBI with 0.1 Gy or 2.0 Gy of X-rays. PBI was performed by exposing the lower third of the mouse body, whilst the upper two thirds were shielded with a shield lead. Under the adopted experimental conditions, for a 2.0 Gy dose at 250 kVp, the dose to the shielded brain was 0.2% of the total dose (4 mGy), demonstrating lack of significant dose contribution to the shielded brain tissues. In this case, 15 days or 6 months post-irradiation the brains were collected for histology (biochemical profiling and neurogenesis analysis) or hippocampus microdissection (miRNA profiling and proteomics).

The exposure system and the overall experimental design scheme are illustrated in Figure 1**.**

**Figure 2:**
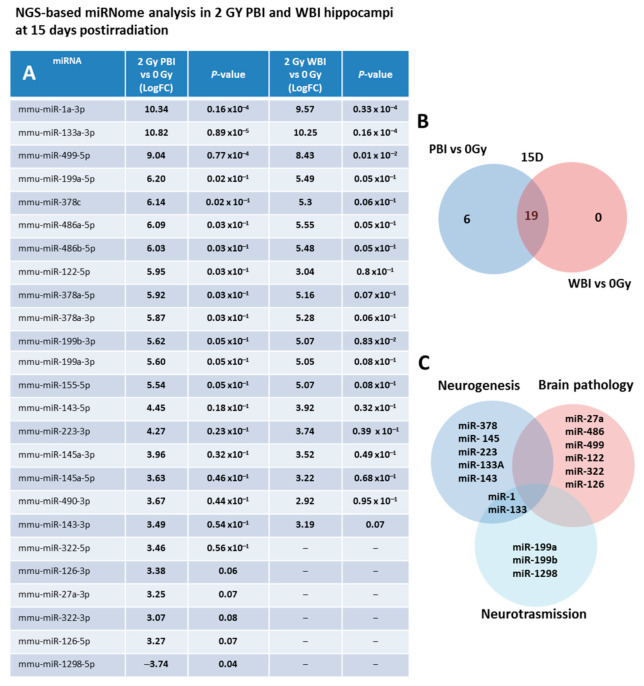
NGS-based miRNAs analysis in 2.0 Gy PBI and WBI hippocampi compared to SI mice at 15 days post-irradiation. (**A**) A *p*-value < 0.1 and a FC > 3 was defined as upregulation; *p*-value < 0.1 and a FC of <3 as downregulation. Samples not meeting the differential expression criteria are in gray. (**B**) Venn diagram of the significantly deregulated and shared genes in the hippocampus of PBI and WBI mice vs. SI mice. (**C**) Analysis of miRNA function. Data shown is from *n* = 3 mice for the SI control, 2 Gy PBI and 2 Gy WBI groups.

As a first step, we investigated the miRNAs perturbation in in-field and out-of-field hippocampi at 15 days post-irradiation through NGS-based miRNome analysis. As a criterion for up-regulation we assumed a *p*-value ≤ 0.1 and a fold change (FC) ≥ 3 and for down-regulation a *p*-value ≤ 0.1 and a FC ≤ −3. Compared to SI hippocampi, miRNome analysis revealed 25 differentially expressed miRNAs in PBI and 19 in WBI mice. In Figure 2A, the miRNAs FC values together with the *P*-values of PBI vs. 0 Gy and WBI vs. 0 Gy are listed. As shown in the Venn diagram (Figure 2B), we detected a marked overlap in miRNAs expression profiles induced by PBI and WBI; all the 19 deregulated miRNAs after WBI were, in fact, also found after PBI. The other 5 miRNAs, (miR-27a-3p, miR-322-3p, miR-322-5p, miR-126-3p, miR-126-5p) over-expressed in PBI group also showed a trend toward increased expression in WBI group but did not fully meet the criteria we set for deregulation. MiR-1298 is unique in two ways: first, it is the only down-regulated miRNA and second it is exclusive for PBI group, being completely unchanged after WBI. As shown in Figure 2C, many of the deregulated miRNAs are involved in brain pathologies being biomarkers of brain injuries (miR-27a, miR-486, miR-499 and miR-122) or associated with neurodegenerative diseases (Parkinson’s and Alzheimer: miR-322 and miR-126); others are involved in the control of neurogenesis (miR-378, miR-145, miR-223, miR 133a, miR-143), neurotransission (miR-1, miR-133, miR-199, miR-1298) or have neuroprotective functions (miR-155). On the complex, our results showing a high degree of similarity in the changes induced by WBI and PBI in miRNA expression profiles are novel and indicate that in-field and out-of-field irradiation cause nearly identical modification in non-coding RNAs in the hippocampus at 15 days post-irradiation.

**Figure 3:**
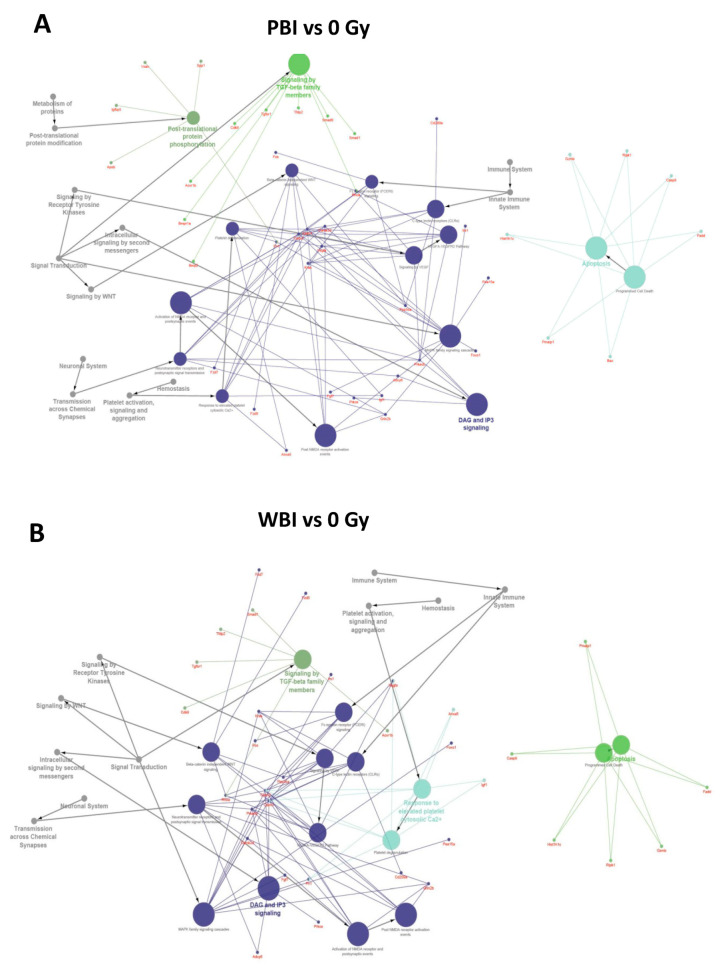
Pathway enrichment analysis of the significantly altered miRNAs in PBI and WBI C57Bl/6 mice compared to SI mice (listed in Figure 2A) obtained using the miRNA enrichment function of Cytoscape plugin CluePedia, selecting the top 20 genes with a miRanda SCORE > 0.6. Focus on some of the predicted genes and corresponding pathways related to the deregulated miRNAs in PBI (**A**) and WBI (**B**).

To further investigate the perturbation induced by PBI and WBI, we searched targets of differentially expressed miRNAs, using the miRNA enrichment function of Cytoscape plugin CluePedia, selecting the top 20 genes with a miRanda SCORE > 0.6. Unsurprisingly, given the high degree of overlapping in deregulated miRNA following WBI and PBI, results of the predicted pathway analysis in the hippocampi 15 days after WBI and PBI converged on the same perturbed regulatory pathways i.e., the Tumor growth Factor (TGF-β) signaling, the apoptotic signaling and the diacylglycerol (DAG) and 1,4,5-trisphosphate [Inositol trisphosphate/calcium (IP3)] signaling, the last one crucial for the transmission across chemical synapses (Figure 3A,B).

**Figure 4:**
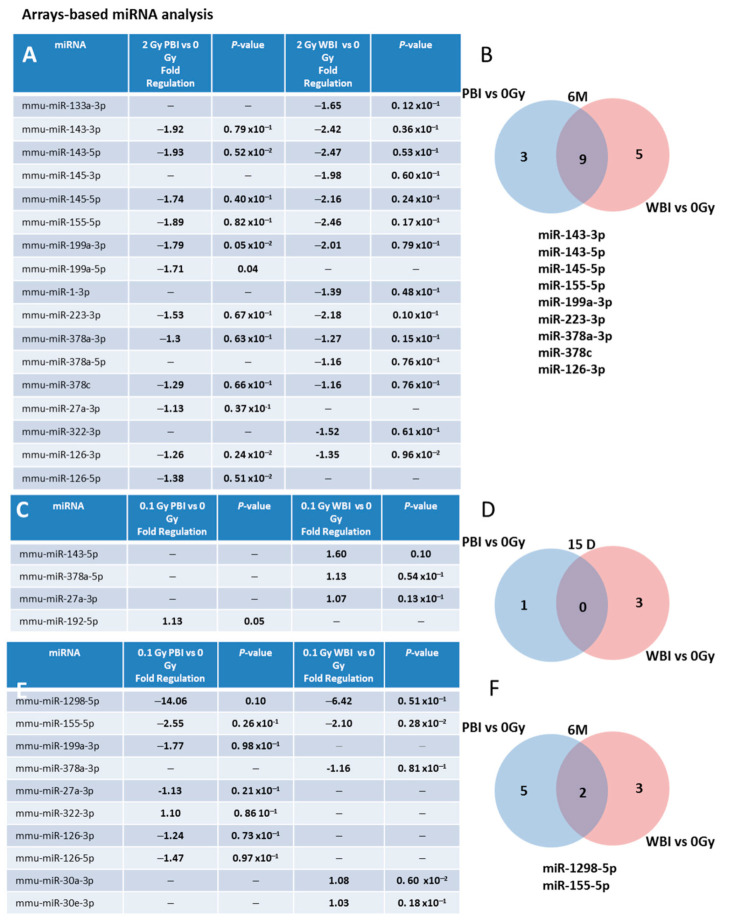
Time- and dose-dependence in radiation-induced modulation of hippocampal miRNAs. (**A**) Hippocampal miRNAs still deregulated 6 months after irradiation with 2 Gy PBI and WBI in custom PCR panels containing the 25 PCR primer sets of the miRNAs found deregulated at 15 days after irradiation with 2.0 Gy PBI. (**B**) Venn diagram of the significantly deregulated and shared genes in the hippocampus of 2.0 Gy PBI and WBI mice vs. SI mice at 6 months post-irradiation. (**C**) Hippocampal miRNAs deregulated 15 days after PBI and WBI exposure with 0.1 Gy in custom PCR panels explained in (**A**). (**D**) Venn diagram of the significantly deregulated and shared genes in the hippocampus of 0.1 Gy PBI and WBI mice vs. SI mice at 15 days post-irradiation. (**E**) Hippocampal miRNAs still deregulated 6 months after exposure at 0.1 Gy PBI and WBI in custom PCR panels explained in (**A**). (**F**) Venn diagram of the significantly deregulated and shared genes in the hippocampus of PBI and WBI mice vs. SI mice at 6 months post-irradiation. Data shown is from *n* = 3 mice for SI control, 2 Gy PBI and 2 Gy WBI at 6 months post-irradiation; SI control, 0.1 Gy PBI, 0.1 Gy WBI at 15 days post-irradition; 0.1 Gy PBI and 0.1 Gy WBI at 6 months post-irradiation.

We also investigated (i) the time-dependence of the modulation of miRNAs induced by exposure at 2.0 Gy in PBI and WBI hippocampi at 6 months post-irradiation and (ii) the time- and dose-dependence in PBI and WBI hippocampi exposed at 0.1 Gy at 15 days or 6 months post-irradiation. To this aim, we developed a miRNA custom PCR arrays ready to use, containing the PCR primer sets of the 25 deregulated miRNAs at 15 days after PBI irradiation with 2.0 Gy. As a criterion for deregulation, we assumed a *p*-value ≤ 0.1. Figure 4A shows the list of deregulated miRNAs 6 months after irradiation with 2.0 Gy. Notably, nine (miR-143-3p, 143-5p, miR-145a-5p, miR-155-5p, miR-199-3p, miR-223-3p, miR-378a-3p, miR-378c and mir-126-3p) of the 19 (47.4%) miRNAs commonly deregulated between PBI and WBI at 15 days after 2.0 Gy irradiation were still commonly deregulated at 6 months post-irradiation, indicating potential long-term health effects of exposure to a moderate radiation dose of 2.0 Gy after PBI or WBI in the hippocampus (Figure 4A,B).

We also analyzed whether PBI or WBI low-dose exposure with 0.1 Gy may perturb the same subset of miRNAs deregulated at 2.0 Gy. At 15 days post-irradiation (Figure 4C,D), we detected only 3 differentially expressed miRNAs in WBI mice (miR-143-5p, miR-378a-5p, miR-27a-3p) and 1 in PBI mice (miR-192-5p), indicating the existence of a dose-response relationship. However, a progressive deregulation of miRNAs was observed in 0.1 Gy irradiated hippocampi at 6 months post-irradiation, with 5 miRNAs deregulated in PBI (miR-199a-3p, miR-27a-3p, miR-322-3p, miR-126-3p, miR-126-5p), 3 in WBI (miR-378a-3p, miR-30a-3p, miR-30e-3p) and 2 miRNAs (miR-1298-5p, miR-155-5p) commonly deregulated between PBI and WBI group (Figure 4E,F). On the whole, opposite time-dependent relationships between the number of deregulated miRNAs and radiation-dose, consisting in decreased miRNAs alteration at 6 months vs. 15 days at higher dose (2.0 Gy), as opposed to progressive perturbation at lower dose (0.1 Gy) were identified after both WBI and PBI.

### 2.2. Effect of in-Field or Out-of-Field Irradiation on the Biochemical Profile of the Hippocampus

**Figure 5:**
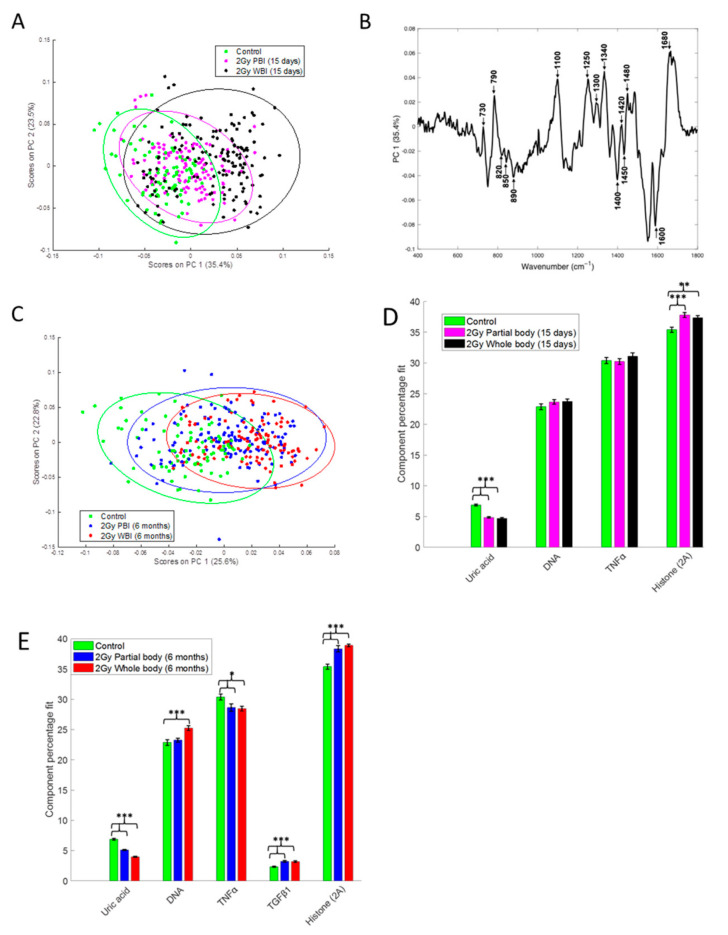
Raman spectral analysis of 2.0 Gy PBI and WBI hippocampi compared to SI control mice at 15 days and 6 months post-irradiation. (**A**) PCA scatterplot of Raman spectral data from control (green), 2.0 Gy PBI (magenta) and 2.0 Gy WBI (black) mice at 15 days post-irradiation. (**B**) PC1 loading from PCA of Raman spectral data from control, 2.0 Gy PBI and 2.0 Gy WBI mice at 15 days post-irradiation showing spectral features responsible for the separation between the groups. (**C**) PCA scatterplot of Raman spectral data from control (green), 2.0 Gy PBI (blue) and 2.0 Gy WBI (red) mice at 6 months post-irradiation. Relative weightings of pure molecular reference species from least squares fit of Raman spectra from (**D**) control, 2.0 Gy PBI and 2.0 Gy WBI groups at 15 days post-irradiation and (**E**) control, 2.0 Gy PBI and 2.0 Gy WBI groups at 6 months post-irradiation. Data shown is from *n* = 5 mice for the SI control, 2 Gy PBI and 2 Gy WBI groups. Error bars represent the standard error. * *p* ≤ 0.05, ** *p* ≤ 0.01 and *** *p* ≤ 0.001.

To compare the response of hippocampus to PBI and WBI we have also used a Raman spectroscopy approach that measures the chemical composition of a sample, allowing the identification of biochemical information. PCA of the Raman spectral data at 15 days after irradiation showed that the 2.0 Gy PBI group appears to be spectrally more similar to the SI group than to the 2.0 Gy WBI group (Figure 5A). The SI and 2 Gy PBI datapoints cluster mainly on the negative side of principal component 1 (PC1), whereas the 2.0 Gy WBI datapoints are mainly clustered on the positive side of PC1. The PC1 loading indicates that nucleic acids (790, 1340, 1480, 1580 cm^−1^), proteins (820, 850, 890, 1450, 1600, 1680 cm^−1^) and lipids (1300, 1400, 1420 cm^−1^) are among the differentiating spectral features (Figure 5B). PCA of the Raman spectral data at 6 months post-irradiation showed that the 2.0 Gy PBI group appears to be spectrally more similar to the 2.0 Gy WBI group than to the SI group (Figure 5C). The 2.0 Gy PBI and 2.0 Gy WBI datapoints cluster mainly on the positive side of PC1, whereas the SI datapoints cluster mainly on the negative side of PC1. The PC1 loading (Figure S1, panel A) is very similar to the PC1 loading shown in Figure 5B for 15 days, indicating that, again, nucleic acids (proteins and lipids) were the main differentiating spectral features. In addition, although overlap of the data is evident, the 0.1 Gy PBI and 0.1 Gy WBI groups could be discriminated from the SI group at 6 months post-irradiation (Figure S1, panels B and C). As before, nucleic acids (proteins and lipids) were found to be the main differentiating spectral features (data not shown).

In addition, CLS fitting analysis was performed to estimate the relative fraction of reference spectra of pure components within the tissue spectra. From the components investigated, DNA, histone 2A, TNFα and uric acid were found to provide the best fit to the tissue spectra at 15 days post-irradiation (Figure 5D) with TGFβ also included for the tissue spectra at 6 months post-irradiation (Figure 5E). Interestingly, histone 2A was found to be significantly increased and uric acid was found to be significantly decreased in the 2.0 Gy PBI and 2.0 Gy WBI groups compared to the SI group at both 15 days and 6 months post-irradiation. In addition, at 6 months post-irradiation, TNFα was found to be significantly decreased and TGFβ was found to be significantly increased in the 2.0 Gy PBI and 2.0 Gy WBI groups compared to the SI group and DNA was found to be significantly increased in the 2.0 Gy WBI group compared to the SI group.

### 2.3. Effect of in-Field or Out-of-Field Irradiation on the Protein Expression in the Hippocampus

**Figure 6:**
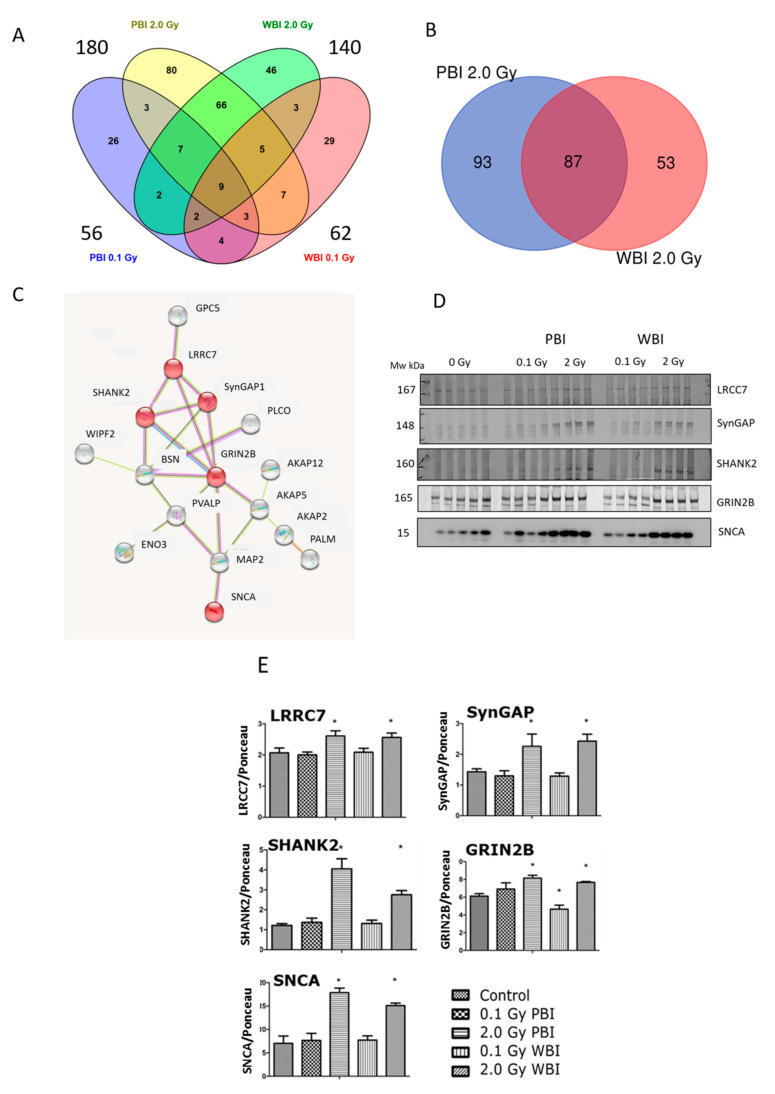
Radiation response of the hippocampal proteome at 15 days post-exposure using 0.1 Gy PBI, 0.1 Gy WBI, 2.0 Gy PBI or 2.0 Gy WBI. (**A**) Venn diagram demonstrating the total numbers of all deregulated proteins in each treatment group, of shared deregulated proteins between the four groups and of proteins exclusively deregulated in each condition (q ≤ 0.05, FC ± 1.3; identification with at least 2 UP, *n* = 4). (**B**) Venn diagram demonstrating the numbers of commonly deregulated and not commonly deregulated proteins in the 2.0 Gy PBI and 2.0 Gy WBI groups. (**C**) Protein–protein interaction analysis using the STRINGdb software tool (http://string-db.org, accessed on 20 April 2021) elucidating a tightly connected cluster consisting of 16 proteins within the commonly deregulated proteins between 2.0 Gy PBI and 2.0 Gy WBI groups. The proteins validated by immunoblotting are indicated in red. The gene names corresponding to the STRINGdb protein symbols are explained in Table 1. (**D**) Immunoblot verification of hippocampal protein changes in different treatment groups. (**E**) The quantification of the immunoblotting results with bar charts representing the average ratios of relative protein expression in control and irradiated samples after background correction to Ponceau. The error bars represent standard error of the mean (+SEM) (*t*-test; * *p* < 0.05; *n* = 4). Data shown is from *n* = 4 mice for all experiments in the SI control, 2 Gy PBI, 2 Gy WBI, 0.1 Gy PBI and 0.1 Gy WBI groups.

Next, a proteomics analysis was performed to gain insight into the out-of-field radiation effect in the hippocampal proteome at 15 days post-irradiation. The proteomics analysis identified 4403 hippocampal proteins in all treatment conditions (Table S1). Proteins fulfilling the following filtering criteria were considered as significantly deregulated: (i) q < 0.05, (ii) identification with at least two unique peptides (UP), (iii) fold change < 0.77 or >1.3. Based on these criteria, the different treatment groups showed the following number of deregulated proteins: 56 (0.1 Gy PBI, Table S2), 62 (0.1 Gy WBI, Table S3), 180 (2.0 Gy PBI Table S4) and 140 (2.0 Gy WBI, Table S5). Of these, only nine proteins were differentially regulated in all groups as shown in the Venn diagram (Figure 6A). In contrast, the hippocampus samples from 2.0 Gy PBI and 2.0 Gy WBI mice had many significantly deregulated proteins in common, altogether 87 (Figure 6B). This corresponded to 48% and 62% of all significantly deregulated proteins in 2.0 Gy PBI and 2.0 Gy WBI groups, respectively. All except two shared proteins showed similar direction of deregulation in PBI and WBI exposure situations, the majority of proteins being upregulated (Table 1). The network analysis elucidated a cluster consisting of 16 proteins (Figure 6C). The majority of these proteins were involved in neurobiological functions such as regulation of long-term neuronal synaptic plasticity and synapse organization (Table S6). Next, some of the expression changes found in the synaptic proteins of the cluster were validated using immunoblotting (Figure 6D, Figure S2). The levels of LRRC7, SynGAP1, SHANK2, GRIN2B and SNCA showed upregulation in the 2.0 Gy-irradiated groups (PBI and WBI) but not in the 0.1 Gy-irradiated groups, similar to the proteomics results (Figure 6E).

**Figure 7:**
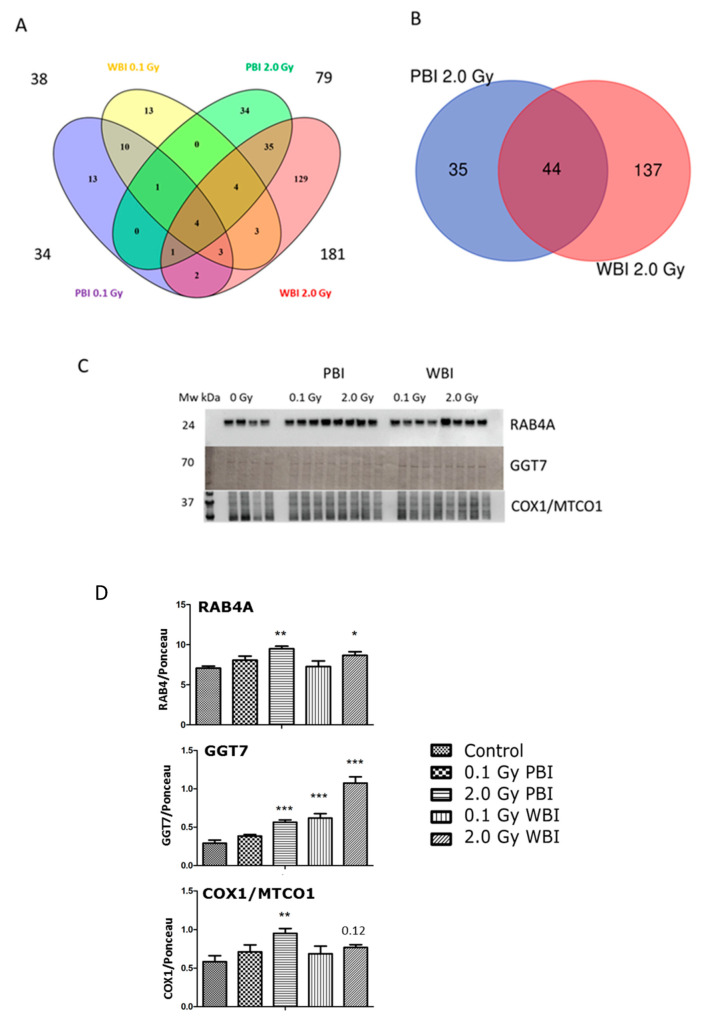
Radiation response of the hippocampal proteome 6 months post-exposure using 0.1 Gy PBI, 0.1 Gy WBI, 2.0 Gy PBI or 2.0 Gy WBI. (**A**) Venn diagram demonstrating the total numbers of all deregulated proteins in each treatment group, of shared deregulated proteins between the four groups, and of proteins exclusively deregulated in each condition (q ≤ 0.05, FC ± 1.3; identification with at least 2 UP, *n* = 4). (**B**) Venn diagram demonstrating the numbers of commonly deregulated and not commonly deregulated proteins in the 2.0 Gy PBI and 2.0 Gy WBI groups. (**C**) Immunoblot verification of hippocampal protein changes in different treatment groups. (**D**) The quantification of the immunoblotting results with bar charts representing the average ratios of relative protein expression in control and irradiated samples after background correction to Ponceau. The error bars represent standard error of the mean (+ SEM) (*t*-test; * *p* < 0.05, ** *p* < 0.01, *** *p* < 0.005; *n* = 4). Data shown is from *n* = 4 mice for all experiments in the SI control, 2 Gy PBI, 2 Gy WBI, 0.1 Gy PBI and 0.1 Gy WBI groups.

The analysis of the hippocampus proteome at 6 months post-irradiation showed fewer differentially regulated proteins than at 2 weeks except for the exposure to 2.0 Gy WBI (Table S7). The different treatment groups showed the following number of deregulated proteins: 34 (0.1 Gy PBI, Table S8), 38 (0.1 Gy WBI, Table S9), 79 (2.0 Gy PBI, Table S10) and 181 (2.0 Gy WBI, Table S11). Of these, only four proteins were differentially regulated in all groups as shown in the Venn diagram (Figure 7A). The 2.0 Gy PBI and 2.0 Gy WBI groups had 44 shared proteins that represents 55.7% and 24.3% of all deregulated proteins in these two groups, respectively (Figure 7B, Figure S3). Many of these deregulated proteins had metabolic functions showing enrichment of the molecular function “catalytic activity” (Figure S3). The 6-month proteomics data were validated using immunoblotting with antibodies against RAB4A, GGT7 and COX1/MTCO1 (Figure 7C,D, Figure S4). In comparison to the SI control, these proteins were upregulated in 2.0 Gy irradiated samples (PBI or WBI) with the exception of COX1/MTCO1 that was significantly upregulated only after 2.0 Gy PBI.

There was only one significantly deregulated protein shared between hippocampus proteomes of 15 days and 6 months following exposure to 0.1 Gy PBI (copine-2) or WBI (integrin beta-2), respectively (Tables S2,S3,S8 and S9). Four proteins (28S ribosomal protein S36, UV excision repair protein RAD23 homolog B, complexin-1 and 60S acidic ribosomal protein P2) were deregulated both at 15 days and 6 months at 2.0 Gy PBI (Tables S4 and S10). The proteome profiles of the hippocampus at 2.0 Gy WBI shared 7 proteins between the two time points (mouse 28S ribosomal protein S36, integrin beta-2, protein sel-1 homolog 1, Ras-related protein Rab-31, mitochondrial import receptor subunit TOM20 homolog, cytochrome c oxidase subunit 7B and claudin-11) (Tables S5 and S11).

Taken together, at 2 weeks, the proteomics data suggested a strong out-of-target effect resembling direct radiation effect in hippocampus at the 2.0 Gy but not at the 0.1 Gy dose, affecting the expression of several synaptic proteins. While the deregulation of synaptic proteins disappeared after 6 months, the proteome response in the 2.0 Gy PBI and WBI groups remained similar, now indicating changes in the level of proteins involved in catabolic activities.

### 2.4. Effect of in-Field or Out-of-Field Irradiation on Adult Hippocampal Neurogenesis

Adult neurogenesis is a multistep process comprising activation of quiescent NSCs, their differentiation into committed progenitor cells, neuronal survival, migration and functional integration of newborn neurons. Several factors have been shown to modulate hippocampal neurogenesis. We and others have shown that hippocampal neurogenesis in rodent models is impaired by ionizing radiation exposures [13,14,15,16,17,18,19,20,21,22].

**Figure 8:**
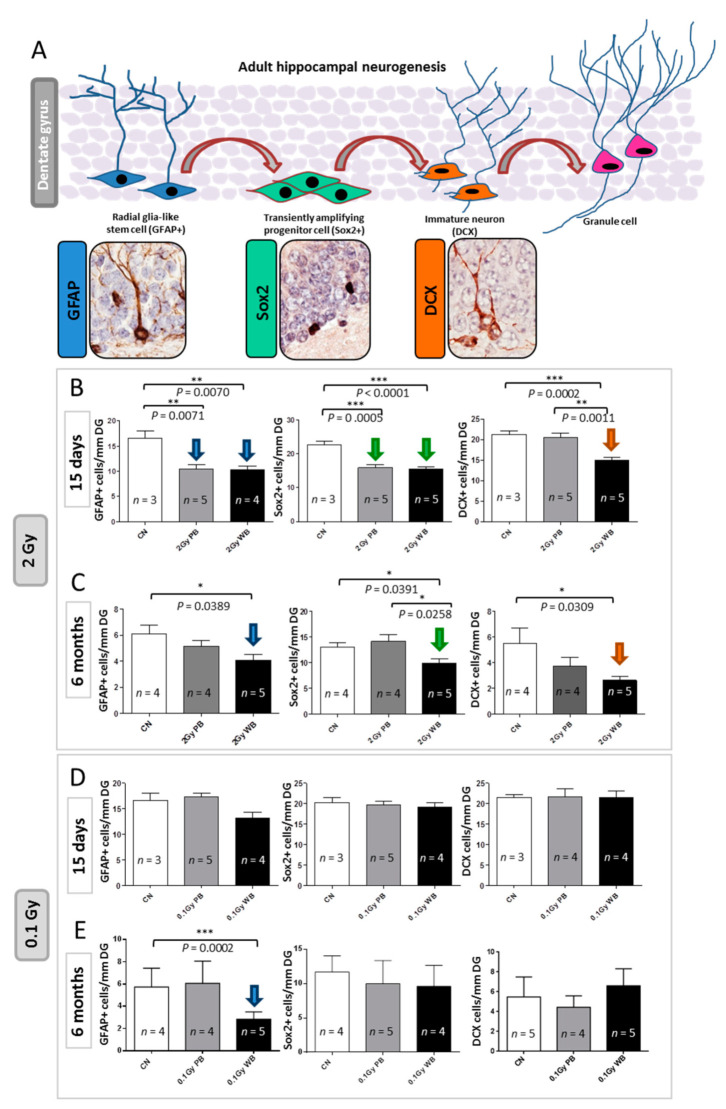
Effect of in-field or out-of-field irradiation on the lineage-specific composition of DG 15 days or 6 months after irradiation with 0.1 and 2.0 Gy of X-rays. (**A**) Schematic representation of adult neurogenesis in the hippocampal DG and relative stage specific markers with representative immunostaining images: glial fibrillary acidic protein (GFAP) for radial glia-like stem cell; sex determining region Y (SRY) box 2 (Sox2) for transient amplifying progenitor cells; doublecortin (DCX) for immature neurons. (**B**) Alteration in the cell stage composition of DG 15 days and (**C**) 6 months following irradiation with 2.0 Gy of X-rays, or (**D**) 15 days and (**E**) 6 months following irradiation with 0.1 Gy of X-rays. The error bars represent standard error of the mean (+SEM) (*t*-test; * *p* < 0.05, ** *p* < 0.01, *** *p* < 0.005).

To test the impact of out-of-field irradiation on hippocampal neurogenesis we evaluated the radiation-dependent modifications in the cellular composition of the SGZ of the DG, through a methodology based on a combination of morphological cellular features and immunohistochemistry with stage-specific neurogenesis markers (Figure 8A). In this case, 15 days after exposure we detected a significant reduction of 37.22% in the number of NSCs labelled by glial fibrillary acidic protein (GFAP) in PBI compared to SI DG (*p* = 0.0071) and a similar significant decrease of 37.70% in WBI (*p* = 0.0070) (Figure 8B). In addition, compared to SI hippocampus, we also observed significant reduction in the percentage of NSC precursors labelled by sex determining region Y (SRY) box 2 (Sox2) of 29.51% (*p* = 0.0005) and 31.65% (*p* = 0.0001) in PBI and WBI respectively. Finally, we observed a significant reduction of 32.77% (*p* = 0.046) in immature neurons labelled by doublecortin (DCX) in the DG of WBI mice but not in PBI mice, representing the unique difference in the defects induced by the two exposure modalities. Altogether, our findings clearly demonstrated that out-of-field irradiation causes defects in the dynamic transition among neural stages in the DG nearly identical to those induced by in-field irradiation 15 days after irradiation with 2.0 Gy. These defects, including self-renewal and proliferation, point to a complex disturbance in the control of NSCs progression into neurons in the hippocampus by out-of-field irradiation. However, while for WBI these defects (i.e., significant depletion of cells labelled by GFAP, Sox2 and DCX) persisted at 6 months post-irradiation, they were transient and were fully recovered at 6 months post-irradiation for PBI exposure (Figure 8C).

Since proteomics and miRNome analysis indicated radiation-induced modulations even at low-dose, we analyzed hippocampal neurogenesis 15 days or 6 months after irradiation with 0.1 Gy. We reported complete lack of functional deficit at 15 days post-irradiation both for WBI and PBI groups (Figure 8D). Instead, at 6 months post-irradiation with 0.1 Gy we detected a significant reduction in the number of NSCs labelled by GFAP in WBI but not in PBI hippocampus (Figure 8E). No difference in the number of precursors labelled by Sox2 and newborn neurons labelled by DCX was detected in both WBI and PBI groups.

## 3. Discussion

Abscopal effects are reported when one part of the animal’s body is exposed to radiation while another part is protected by a lead shield [23,24,25,26].

Very little is known about the impact of abscopal effects on a shielded brain upon the irradiation of distal organs. While our pioneering work highlighted the importance of communication between irradiated and non-irradiated tissues/organs in cancer induction in vivo [7,8], the potential contribution of out-of-target radiation effects in non-cancer pathologies is still scarcely investigated. Radiation-induced damage to the hippocampus, is known as a major determinant in cognitive dysfunction [4,27,28,29,30]. Here we have employed a multi-omic approach to provide a greater and comprehensive understanding of the sequence of events leading to radiation injury in the hippocampus, dissecting targeted and non-targeted radiation responses. To this aim, to integrate the information from multiple layers of biological data, we carried out miRNome, proteomics and biochemical profiling and analysis of adult hippocampal neurogenesis function.

Our findings demonstrated here, for the first time, that 15 days following exposures of the lower third of the mouse body with 2.0 Gy of X-rays, the shielded hippocampus exhibited changes in miRNA and protein profiles nearly identical to those induced by WBI with the same dose. In strict concordance, the analysis of hippocampal neurogenesis at 15 days after irradiation demonstrated marked defects in the dynamic transition among neural stages, mainly involving NSCs and progenitors in the DG, nearly identical after PBI and WBI, suggesting that in-field and out-of-field irradiation induce very similar disturbance in the control of progression of NSCs into neurons in the hippocampus.

Notwithstanding the profuse investigations there is still much to learn about the magnitude, the molecular mechanisms and the consequences of RIBE on the brain and their contributions to the side effects of radiation therapy. Evidence suggests that non-targeted effects in non-irradiated cells may be mediated via cell-to-cell gap junctions (GJ) and through mediators released from irradiated cells, especially cytokines and chemokines [31,32]. Indeed, the unique exclusive miRNAs of the PBI group was mir-1298, which has been reported to act as a negative regulator of the GJ protein Cx43, by facilitating degradation of *Cx43* mRNA through specific binding [33]. Therefore, mir-1298 down-regulation in PBI hippocampus, by increasing Cx43 protein level, may enhance intercellular communication facilitating the propagation of damage-signals from irradiated tissues. An involvement of Cx43 in the long-range transmission of bystander signals is also supported by our previous findings, demonstrating that GJ is critical for radiation-associated bystander tumorigenesis in the central nervous system in a mouse model with *Cx43* deletion in which radiation-induced out-of-target tumorigenesis is drastically reduced [8].

Other miRNAs, here deregulated in response to irradiation, are involved in cellular trafficking and communication, suggesting that both PBI and WBI may target cellular trafficking in the hippocampus. Mir-1 is the top upregulated miRNA in our settings and its overexpression in the heart has been reported to attenuate hippocampal synaptic vesicle exocytosis by the posttranscriptional regulation of SNAP-25, through the transportation of exosomes [34]. Additionally, mir-199a/b family, which we found markedly upregulated following irradiation, is involved in the control of multiple endocytosis related genes [35]. Therefore, defects in the membrane trafficking, a hallmark of many neurodegenerative disorders, may concur to the pathogenesis of hippocampal radiation-damage.

Activation of cytokines has been associated with abscopal radiation effect and macrophage activation, followed by a storm of cytokines including IL-1a, IL-1b, IL-6, TNF-α and TGF-β, accompanied the induction of abscopal radiation effects [36,37,38]. In strong agreement, our miRNA-based predicted pathway analysis identified the TGF-β signaling among the perturbed regulatory pathways in the hippocampi at 15 days after WBI and PBI, strongly implying the TGF-β signaling in targeted and non-targeted radiation-responses. Notably, the TGF-β superfamily cytokines are principal regulators of adult hippocampal neurogenesis [39] controlling proliferation, differentiation, maturation and survival of NSCs and precursors in the neurogenic niches of the adult brain [40]. TGB-β is also involved in changes in neurogenesis in response to injury [41] and may convey stimulatory or inhibitory responses depending on the neuronal cell type [42]. Therefore, radiation-induced perturbation of the TGF-β signaling may be responsible of the alterations detected in hippocampal neurogenesis after irradiation. In addition, we found that radiation-induced perturbation in NSCs self-renewal in the DG is associated with up-regulation of master miRNAs of the dynamic control of stem cell homeostasis, such as miR-378 and miR-145. Both are well-known regulators of NSCs self-renewal; miR-378 has a target site in the 3′-untranslated region of Tailless (TLX) [43] and miR-145 directly downregulates Sox2 [44]. Therefore, although a causative link cannot be established, it is likely that radiation-dependent upregulation of miR-378 and miR-145 influences the progression of NSCs into neurons, supporting a critical role for these miRNAs as regulators of neurogenesis after injury.

Additionally, DAG and IP3 signaling pathways are among the predicted perturbed regulatory pathways at 15 days after WBI and PBI. IP3 pathway, is involved in brain development, axonal growth, memory formation and excitability, and its deregulation contributes to the onset of many neurodegenerative diseases including Alzheimer, Amyotrophic lateral sclerosis and Autism spectrum disorders [45]. DAG is an important signaling lipid molecule at neuronal synapses and it has been implicated in various form of synaptic plasticity, including hippocampal long-term potentiation [46]. Therefore, alterations of the DAG and IP3 signaling pathways are also likely to contribute to radiation-induced effects in the hippocampus.

Crucial for full neuronal functionality is the ability to modulate and adjust its proteome in response to specific cues, for example, synaptic remodeling in response to patterns of action potentials. Neuronal plasticity is the result of a balance between protein synthesis and degradation to maintain and regulate synaptic protein turnover. Significant upregulation in a subset of synaptic protein, LRRC7, SynGAP1, SHANK2, GRIN2B and SNCA, experimentally validated, pointed to important synaptic dysfunction, a prominent feature of many neuropathological conditions. Long-term proteomic changes involving synaptic plasticity have already been reported in our previous work following cranial or whole-body irradiation of in utero or neonatal mice [13,47,48,49]. The novel aspect here is that the hippocampus of mice irradiated with the upper two thirds of the body shielded share the same proteomic changes of directly irradiated hippocampus, relating to long-term neuronal synaptic plasticity and synapse organization.

The Raman spectral data indicate the occurrence of progressive radiation-induced metabolic changes in PBI hippocampus revealed by the biochemical similarity of 2.0 Gy PBI and 2.0 Gy WBI groups at 6 months post-irradiation, while at 15 days post-irradiation the 2.0 Gy PBI group overlapped with the SI group. The CLS analysis showed significantly differentiated molecular species in the 2.0 Gy PBI and 2.0 Gy WBI groups compared to the control group including histone 2A, DNA, TNFα, TGFβ and uric acid. The proteomics analysis also found histone 2A to be significantly deregulated in the 2.0 Gy PBI and 2.0 Gy WBI groups and the miRNome analysis indicated involvement of the TGFβ signaling pathway. Cytokines, such as TNFα and TGF-β and uric acid, are considered to be danger signals released from dead or damaged cells in response to radiation [50,51].

### 3.1. Time-Dependence of Radiation Responses after WBI and PBI with 2.0 Gy

**Figure 9:**
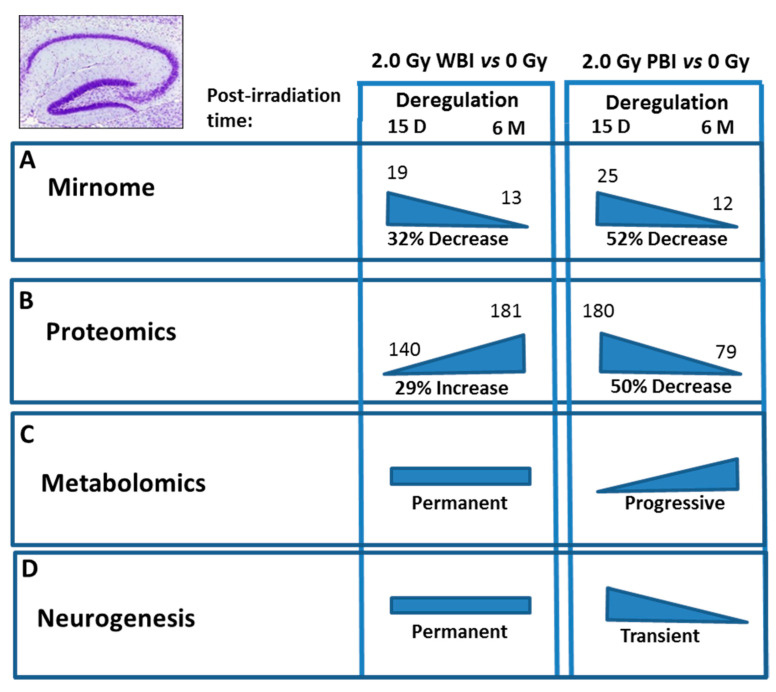
Summary of the time-dependence for multiple endpoints in the hippocampus after WBI and PBI with 2.0 Gy of X-rays. (**A**) Deregulated miRNAs showed a 32% decrease at 6 months (13) vs. 15 days (19) in WBI group and a 52% decrease in age-matching PBI group (12 vs. 25). (**B**) Proteomics showed a 29% increase in the number of deregulated proteins at 6 months (181) vs. 15 days (140) in WBI group and a 50% decrease in in age-matching PBI group (79 vs. 180). (**C**) Raman spectroscopy at 15 days post-irradiation showed that only the spectral fingerprints of WBI differed from that of SI hippocampi while PBI and SI could not be discriminated. At 6 months post-irradiation the WBI and PBI hippocampi could not be discriminated and both differed from SI one. (**D**) Neurogenesis data showed long lasting defects in WBI mice at 6 months post-irradiation, while all the defects observed at 15 days post-irradiation in PBI had disappeared at 6 months post-irradiation. Altogether, the majority of data indicated the transitory nature of the PBI effects compared to the persistence of the WBI induced-responses.

Comparison of the effects at 15 days and 6 months post-irradiation allows the evaluation of the time-dependence of radiation responses after WBI and PBI with 2.0 Gy (Figure 9) suggesting an attenuation of bystander response with time. Deregulated miRNAs showed a consistent decrease of 52% (12 vs. 25) in PBI groups at 6 months vs. 15 days, and a smaller decrease of 31.6% (13 vs. 19) in age-matching WBI groups. Similarly, proteomics showed a marked time-dependent decrease of around 50% in the number of deregulated proteins in PBI groups (79 vs. 180) at 6 months vs. 15 days, as opposed to the increase in the number of deregulated proteins in age-matching WBI groups (181 vs. 140). In addition, the alteration in the synaptic protein network disappeared at 6 months both in PBI and WBI groups, when the deregulation affected, instead, proteins involved in catabolic activities both for PBI and WBI. Accordingly, with the above results, pointing to a persistence of molecular alterations in the hippocampus of WBI mice, the analysis of neurogenesis showed that the totality of defects detected at 15 days post-irradiation had disappeared with time in PBI but not WBI hippocampus. Raman spectroscopy, by showing that at 15 days post-irradiation the spectral fingerprints of the 2.0 Gy-PBI and SI hippocampus have strong similarities and cannot be discriminated, while at 6 months post-irradiation the 2.0 Gy-PBI could not be discriminated from 2.0 Gy-WBI and both differed from SI ones, seems to suggest slow progressing radiation-induced metabolic changes after PBI. Altogether, the great majority of data (miRNA, proteomics and neurogenesis) indicated the transitory nature of the PBI effects compared to the persistence of the WBI induced-responses, clearly pointing to important mechanistic differences between direct and out-of-field radiation responses to be further explored.

### 3.2. Dose-Dependence of Radiation Responses after WBI and PBI with 0.1 Gy

Investigation on dose-dependence of radiation responses in the hippocampus following WBI and PBI with 0.1 Gy showed the existence of a dose-response relationship. In fact, 15 days post-irradiation with 0.1 Gy a very small number of miRNAs was modulated both after PBI (*n* = 1) and WBI (*n* = 3) with no commonly deregulated miRNAs. In addition, the number of deregulated proteins showed a strong reduction compared to the deregulation level induced at 2.0 Gy, with lack of involvement of synaptic proteins. Concordantly, analysis of neurogenesis indicated that 15 days after PBI and WBI with 0.1 Gy effects could be detected compared to the SI controls. Altogether, these data revealed only small effects at early post-irradiation times after low-dose irradiation with 0.1 Gy. Instead, the consequences of low-dose irradiation with 0.1 Gy were generally worsening with the time progressing. Biochemical profiles, for instance, indicated clear spectral differences between SI control and PBI or WBI samples at 6 months post-irradiation. In addition, the analysis of neurogenesis showed defects, although only in WBI hippocampi, consisting in a significant decrease in the pool of NSCs compartment labelled by GFAP. At 6 months post-irradiation with 0.1 Gy, we also observed an increase in the number of deregulated miRNAs that included a marked deregulation of mir-1298, controlling the GJ protein Cx43 both in PBI and WBI. Finally, we also identified miR-155 as commonly deregulated in PBI and WBI mice at 6 months after irradiation with 0.1 Gy and at both 15 days and 6 months after 2.0 Gy exposure, supporting its functional role in radiation responses. miR-155 is pro-inflammatory factor in a variety of organ systems and it is strongly upregulated following brain injuries, although whether it is beneficial [52] or detrimental [53] is still controversial. On the complex, our data demonstrated a dose-dependent- related differences in the timing of manifestation of defect in the hippocampus.

## 4. Materials and Methods

### 4.1. Animal Irradiation

C57Bl/6J female mice of 8 weeks of age were subjected to WBI or PBI with 0.1 or 2.0 Gy of X-rays. Irradiation was performed using a Gilardoni CHF 320 G X-ray generator (Gilardoni, Mandello del Lario, Italy) operated at 250 kVp, 1 mA for 0.1 Gy and 15 mA for 2.0 Gy, with Half-Value Layer = 1.6 mm Cu (additional filtration of 2.0 mm Al and 0.5 mm Cu). PBI was performed by exposing the lower third of the mouse body, whilst the upper two thirds were shielded with a lead-shield. Additional groups of mice were sham irradiated (SI).

### 4.2. Dosimetry

A dosimetric evaluation approach, based on experimental measurements with an ionization chamber NE 2571, had been employed to evaluate the dose to the shielded brain resulting from photons crossing the leads shield or deflected in the cap through the irradiated tissue. Under the adopted experimental conditions, there was no significant dose contribution to the shielded brain (Supplementary Dosimetric Information).

### 4.3. RNA Isolation, Library Preparation and Next Generation Sequencing (NGS)

In this case, 15 days after irradiation, PBI, WBI and SI hippocampi were collected and total RNA was extracted using miRNeasy kit (≠217,004; QIAGEN, Milan, Italy) according to the manufacturer’s instructions. Total RNA (1 μg) was converted into miRNA NGS libraries using NEBNEXT library generation kit (New England Biolabs Inc., Beverly, MA, USA) following manufacturer’s instructions. Samples were sequenced on the Illumina NextSeq 500 System. All sequencing data analysis was performed using the R platform (http://www.r-project.org (accessed on 28 November 2018)) and the open-source Bioconductor libraries. Data were filtered based on sequence counts (i.e., >1 reads per million in at least 2 samples) and pairwise comparisons of differential miRNA expression were performed using edgeR package [54,55,56]. MiRNAs with a *p*-value < 0.1 were used for gene/miRNA enrichment analysis with Cytoscape plug-in “ClueGo” (v.2.1.7) and “CluePedia” (v.1.1.7) [57]. For each miRNA list, enrichment was performed for individual miRNAs employing the miRanda database (miRanda score threshold = 0.6) and showing the top 20 predicted target genes corresponding to each miRNA. Subsequently, predicted target genes and miRNAs were selected to find the affected functions on the Reactome database [58,59].

### 4.4. miRNA Custom PCR Arrays

A miRNA custom PCR arrays ready to use (miRCURY LNATM miRNA Custom PCR Panel PCR Panel YCA22491, QIAGEN, Milan, Italy), containing the 25 PCR primer sets of deregulated miRNAs at 15 days after irradiation with 2.0 Gy PBI, was developed. Total RNA was used as starting material for the procedure of detection and quantification of miRNA expression. Reverse transcription of total hippocampal RNA was performed with miRCURY-LNA-RT-Kit according to the manufacturer’s instructions (Cat 339340, QIAGEN, Milan, Italy). As a criterion for deregulation we assumed a *p*-value ≤ 0.1.

### 4.5. Immunohistochemistry

Fixed tissue sections were immunostained as described [13] using the following primary antibodies: GFAP (Z0334, Dako, Carpinteria, CA, USA; 1:500), Sox2 (ab97959, Abcam, Cambridge, UK; 1:500) and DCX (18723, Abcam; 1:2000). Cell quantification was performed on collected sections (stained for GFAP, Sox2 and DCX) using the imaging software NIS-Elements BR 4.00.05 (Nikon Instruments Europe B.V., Firenze, Italy). The number of positive cells in the subgranular zone (SGZ) were expressed per mm of the SGZ length. Neural stem cells (NSCs) were counted based on criteria including SGZ localization, positive labeling and morphology. Statistical significance was determined using a two-tailed student’s *t*-test for comparison between pairs of means. *p*-values ≤ 0.05 were considered to be statistically significant.

### 4.6. Raman Spectroscopy

OCT embedded brain tissue was sectioned (10 μm) using a cryostat. An XploRA confocal Raman instrument (HORIBA Jobin Yvon, Edison Township, NJ, USA) was used for spectral acquisition. Manual calibration of the grating was carried out using the 520.7 cm^−1^ Raman line of crystalline silicon. Dark current measurement and recording of the substrate and optics signal was also performed, for data correction. As source, a 532 nm laser of ~12 mW power was focused by a 100× objective (MPlanN, Olympus, Shinjuku-ku, Japan, NA = 0.9) onto the sample and the resultant Raman signals were detected using a spectrograph with a 1200 g/mm grating coupled with a CCD. Raman spectra were acquired in the 400 to 1800 cm^−1^ region with an integration time of 30 s per spectrum and averaged over two accumulations. Multiple calibration spectra of 1,4-Bis(2-methylstyryl)benzene were recorded along with each sample acquisition. All spectra were subsequently wavenumber calibrated using in-house developed procedures in Matlab v.9.3 (Mathworks Inc., Natick, MA, USA). The instrument response correction was performed using the spectrum of NIST Standard Reference Material (SRM) no.2242. Spectra were recorded from the hippocampus region of the brain from 5 individual mice per group.

#### Data Pre-Processing and Analysis

All spectral processing procedures were conducted using Matlab (R2017a; Mathworks Inc., Natick, MA, USA), along with in-house developed algorithms and procedures available within the PLS Toolbox (v 8.0.2, Eigenvector Research Inc., Wenatchee, MA, USA). Briefly, spectra were imported, baseline was subtracted with a rubberband algorithm, vector normalized and smoothed using a Savitzky-Golay smoothing algorithm with a 7-point window and a 5th order polynomial.

Subsequently, corrected spectra were subjected to Principal Components Analysis (PCA). In brief, PCA is a commonly used method for multivariate data reduction and visualization. It is an unsupervised method used to describe variance in data sets by identifying a new set of orthogonal features, called principal components (PCs).

Classical least squares (CLS) fitting analysis was performed as described previously [60,61,62] using a set of reference spectra (set: Actin, Apolipoproteins, ATP, Beta-Carotene, Ceramide, Clusterin, Cytochrome C, Cholesterol, Creatinine, DNA, Epidermal growth factor, Glucose, Histone 2A, Interleukins, Keratinocyte growth factor, Phosphatidyl choline, Polyunsaturated fatty acids, TGF-β1, Tumor necrosis factor (TNF)-α and Uric acid) of pure molecular species which were purchased from Sigma-Aldrich (Wicklow, Ireland) In brief, CLS is an exploratory method that aims to minimize the squared differences between the fit and the spectrum using a set of reference pure molecular spectra. It assumes that any complex spectrum, *S*, is the linear sum of contributions from spectra of pure components, *a*_1_, *a*_2_, … *a_n_*, that contribute to the spectrum as follows [63]:

*S* = *a*_1_*C*_1_+ + *a*_2_*C*_2_…*E* (1)

where *C*_1_, *C*_2_,… *C_n_*, are the weights or concentrations assigned to each component spectrum. In the case of a Raman spectrum, not all contributing pure components are known. Therefore, *E* represents the error or residual matrix.

### 4.7. Proteomics Analysis

#### 4.7.1. Sample Preparation for Proteomics

Each 10 µg protein sample in 0.1x RIPA buffer was subjected to tryptic digest applying a modified filter-aided sample preparation (FASP) procedure [64,65]. After protein reduction and alkylation using DTT and iodoacetamide, samples were denatured in UA buffer (8 M urea in 0.1 M Tris/HCl pH 8.5), centrifuged on a 30 kDa cut-off filter device (Sartorius) and washed three times with UA buffer and twice with 50 mM ammoniumbicarbonate (ABC). Proteins were lysed for 2 h at room temperature using 0.5 µg Lys-C (Wako Chemicals, Neuss, Germany) and subsequently for 16 h at 37 °C using 1 µg trypsin (Promega, Mannheim, Germany). Peptides were collected by centrifugation (10 min at 14,000× *g*) and acidified with 0.5% trifluoroacetic acid (TFA) and stored at −20 °C.

#### 4.7.2. Mass Spectrometric Measurements

LC-MSMS analysis was performed in data-dependent acquisition (DDA) mode. MS data were acquired on a Q-Exactive HF-X mass spectrometer (Thermo Fisher Scientific, Waltham, MA, USA) each online coupled to a nano-RSLC (Ultimate 3000 RSLC; Dionex). Tryptic peptides were automatically loaded on a C18 trap column (300 µm inner diameter (ID) × 5 mm, Acclaim PepMap100 C18, 5 µm, 100 Å, LC Packings) at 30 µL/min flow rate. For chromatography, a C18 reversed phase analytical column (nanoEase MZ HSS T3 Column, 100 Å, 1.8 µm, 75 µm × 250 mm, Waters) at 250 nL/min flow rate in a 95 min non-linear acetonitrile gradient from 3% to 40% in 0.1% formic acid was used. The high-resolution (60,000 full-width at half-maximum) MS spectrum was acquired with a mass range from 300 to 1500 *m*/*z* with automatic gain control target set to 3 × 10^6^ and a maximum of 30 ms injection time. From the MS pre-scan, the 15 most abundant peptide ions were selected for fragmentation (MSMS) if at least doubly charged, with a dynamic exclusion of 30 s. MSMS spectra were recorded at 15,000 resolution with automatic gain control target set to 5 × 10^2^ and a maximum of 50 ms injection time. The normalized collision energy was 28 and the spectra were recorded in profile mode.

#### 4.7.3. Progenesis QI Analysis for Label-Free Quantification for 6 Months Data

Spectra were analyzed using Progenesis QI software for proteomics (Version 4, Nonlinear Dynamics, Waters, Newcastle upon Tyne, UK) for label-free quantification as previously described [64] with the following changes: Spectra were searched against the Swissprot mouse database (Release 2017_02, 16,872 sequences). Search parameters used were 10 ppm peptide mass tolerance and 20 mmu fragment mass tolerance. Carbamidomethylation of cysteine was set as fixed modification and oxidation of methionine and deamidation of asparagine and glutamine was allowed as variable modifications, allowing only 1 missed cleavage site. Mascot integrated decoy database search was set to a false discovery rate (FDR) of 1% with a percolator ion score cut-off of 13 and an appropriate significance threshold. Peptide assignments were imported into Progenesis QI. Normalized abundances of peptides were summed up and allocated to the respective protein. Statistical analysis of differences between experimental groups was performed in QI using ANOVA generating *P*-values as well as q-values based on an optimized FDR approach.

#### 4.7.4. Proteome Discoverer Analysis for Analysis at 15 Days Post-Irradiation

Proteome Discoverer 2.4 software (Thermo Fisher Scientific; version 2.4.1.15) was used for peptide and protein identification via a database search (Sequest HT search engine) against SwissProt mouse data base (Release 2020_02, 17,061 sequences), considering full tryptic specificity, allowing for up to two missed tryptic cleavage sites, precursor mass tolerance 10 ppm, fragment mass tolerance 0.02 Da. Carbamidomethylation of Cys was set as a static modification. Dynamic modifications included deamidation of Asn and Gln, oxidation of Met; and a combination of Met loss with acetylation on protein N-terminus. Percolator was used for validating peptide spectrum matches and peptides, accepting only the top-scoring hit for each spectrum and satisfying the cutoff values for FDR < 1%, and posterior error probability <0.01. The final list of proteins complied with the strict parsimony principle.

The quantification of proteins was based on abundance values based on the area for unique plus razor peptides. Abundance values were normalized in a retention time dependent manner to account for sample loading errors. The protein abundances were calculated summing up the abundance values for admissible peptides. The final protein ratio was calculated using median abundance values of 4 to 5 replicate analyses each and defined to be up to 100. The statistical significance of the ratio change was ascertained employing the *t*-test approach described in [66] which is based on the presumption that we look for expression changes for proteins that are just a few in comparison to the number of total proteins being quantified. The quantification variability of the non-changing “background” proteins can be used to infer which proteins change their expression in a statistically significant manner. P-values are adjusted according the method of Benjamini-Hochberg. The analyses of protein-protein interaction and signaling networks were performed by the software tools INGENUITY Pathway Analysis (IPA) (Qiagen, Inc., Hilden, Germany, https://www.qiagenbioinformatics.com/products/ingenuity-pathway-analysis [67], accessed on 20 April 2021).

### 4.8. Immunoblotting

The immunoblotting was performed as described previously [68]. The following antibodies were used: anti-LRRC7 (PA5-70660; Thermo Fisher Scientific), anti-SHANK2 (#12218; Cell Signaling Technology, Beverly, MA, USA), anti-GRIN2B (06-600; Merck Millipore, Burlington, MA, USA), anti-SynGAP (#3200; Cell Signaling Technology), anti-Alpha-Synuclein (610787; BD Biosciences, Franklin Lakes, NJ, USA), anti-RAB4A (ab13252; Abcam), anti-GGT7 (ab80903, Abcam) and anti-MTCO1 (ab45918, Abcam).

### 4.9. Statistical Analysis

For proteomics, the following filtering criteria were used: proteins identified and quantified with at least two unique peptides (UP), had a q-value of ≤0.05 and fold-changes of ≤0.77 or ≥1.3 were considered as significantly differentially expressed. Six biological replicates per treatment group were included in the analysis.

For immunoblotting, statistical analysis was performed with Graph Pad prism software (GraphPad Software, San Diego, CA, USA) using an unpaired Student’s *t* test. The error bars represent standard error of the mean (±SEM) (*t*-test; * *p* < 0.05; *n* = 3). Each treatment group was compared individually to the sham-irradiated control group.

### 4.10. Data Availability

The mass spectrometry proteomics data have been deposited to the ProteomeXchange Consortium via the PRIDE [69] partner repository with the dataset identifier PXD024975.

## 5. Conclusions

In summary, we successfully identified molecular alterations in non-coding RNAs, proteins and metabolic levels as well as histological changes in the rate of hippocampal neurogenesis following PBI and WBI, clearly demonstrating the existence of non-targeted radiation effects in mouse hippocampus. Predicted pathway analysis identified alterations in DAG/IP3 and TGF-β signaling pathways as well as changes in the expression level of proteins involved in the regulation of long-term neuronal synaptic plasticity and synapse organization, coupled with defects in NSCs self-renewal in the hippocampus. At moderate doses (2.0 Gy) the majority of the PBI effects were transient, well detectable at early post-irradiation times and decreasing at 6 months post-irradiation, indicating mechanistic difference with the long-lasting WBI effects. The opposite was observed at low dose (0.1 Gy), with a progressive accumulation of cellular and molecular defects becoming more manifested at 6 months post-irradiation.

Even though our mechanistic observations are referred to a mouse model, our conclusions, emphasizing that both targeted and non-targeted radiation effects play a role in the pathogenesis of hippocampal radiation-damage, might have more general implications for human health. For instance, region-sparing during radiotherapy, aimed at dose reduction to regions of neurogenesis, may be less effective than expected due to of out-of-target effects coming from other irradiated tissue/organs. Elucidating the pathogenic biological and molecular mechanism underlying out-of-target radiation effects in the brain is relevant for prevention of radiation-induced brain injury and optimization of therapeutic strategies for the treatment of damage stemming from radiotherapy.

## Figures and Tables

**Table 1:** List of commonly deregulated hippocampal proteins showing significantly changed expression 15 days after 2.0 Gy PBI and 2.0 Gy WBI.

Accession	Description	Gene Symbol	FC PBI 2 Gy	FC WBI 2 Gy
Q8BRV5	Uncharacterized protein KIAA1671	2900026A02Rik	2.331	1.792
Q0VBF8	Protein stum homolog	6330403A02Rik	1.722	1.353
Q8N9S3	Activator of 90 kDa heat shock protein ATPase homolog 2	Ahsa2	2.179	1.825
Q9WTQ5	A-kinase anchor protein 12	Akap12	1.756	1.510
O54931	A-kinase anchor protein 2	Akap2	1.682	1.550
D3YVF0	A-kinase anchor protein 5	Akap5	1.652	1.544
Q91W96	Anaphase-promoting complex subunit 4	Anapc4	0.010	0.227
P97384	Annexin A11	Anxa11	0.480	0.558
Q07076	Annexin A7	Anxa7	0.659	0.427
Q9Z1R2	Large proline-rich protein BAG6	Bag6	1.383	1.360
Q3UNZ8	Quinone oxidoreductase-like protein 2	BC026585; Cryzl2	1.607	0.446
Q80YN3	Breast carcinoma-amplified sequence 1 homolog	Bcas1	1.931	1.481
O88737	Protein bassoon	Bsn	1.585	1.341
Q9D8X2	Coiled-coil domain-containing protein 124	Ccdc124	1.476	1.398
Q9WU84	Copper chaperone for superoxide dismutase	Ccs	0.071	7.561
Q4VAA2	Protein CDV3	Cdv3	1.672	1.675
Q9JKC6	Cell cycle exit and neuronal differentiation protein 1	Cend1	2.763	1.347
Q6A065	Centrosomal protein of 170 kDa	Cep170	1.656	1.377
Q9D1L0	Coiled-coil-helix-coiled-coil-helix domain-containing protein 2	Chchd2	2.336	2.430
Q8VEA4	Mitochondrial intermembrane space import and assembly protein 40	Chchd4	1.538	1.580
P53996	Cellular nucleic acid-binding protein	Cnbp	0.701	0.597
Q80WW9	DDRGK domain-containing protein 1	Ddrgk1	2.049	2.428
Q8K382	DENN domain-containing protein 1A	Dennd1a	2.291	2.165
Q61495	Desmoglein-1-alpha	Dsg1a	0.316	0.253
P57776	Elongation factor 1-delta	Eef1d	1.324	1.343
Q3UMY5	Echinoderm microtubule-associated protein-like 4	Eml4	100	100
P21550	Beta-enolase	Eno3	1.927	2.079
Q8BJH1	Zinc finger C2HC domain-containing protein 1A	Fam164a; Zc2hc1a	1.755	1.44
P06880	Somatotropin	Gh	0.174	0.359
Q8CAL5	Glypican-5	Gpc5	0.600	0.522
Q3UNH4	G protein-regulated inducer of neurite outgrowth 1	Gprin1	3.659	2.216
Q01097	Glutamate receptor ionotropic. NMDA 2B	Grin2b	1.421	1.366
Q3THW5	Histone H2A.V	H2afv	0.751	0.613
P01942	Hemoglobin subunit alpha	Hba-a1; Hba-a2	2.626	4.491
P02088	Hemoglobin subunit beta-1	Hbb-b1	2.318	4.502
Q9CQ22	Ragulator complex protein LAMTOR1	Lamtor1	3.583	2.941
Q61792	LIM and SH3 domain protein 1	Lasp1	1.967	1.416
Q3UN02	Lysocardiolipin acyltransferase 1	Lclat1	0.660	0.674
Q80TE7	Leucine-rich repeat-containing protein 7	Lrrc7	1.457	1.532
Q9QXA5	U6 snRNA-associated Sm-like protein LSm4	Lsm4	1.881	1.807
Q9QYR6	Microtubule-associated protein 1A	Map1a; Mtap1a	2.101	1.616
P20357	Microtubule-associated protein 2	Map2; Mtap2	1.729	1.495
Q7TSJ2	Microtubule-associated protein 6	Map6; Mtap6	2.872	2.019
A2AJI0	MAP7 domain-containing protein 1	Map7d1; Mtap7d1	1.870	1.458
A2AG50	MAP7 domain-containing protein 2	Map7d2; Mtap7d2	1.746	1.652
B1AUR6	Protein MMS22-like	Mms22l	1.566	0.586
Q9CQX8	28S ribosomal protein S36. mitochondrial	Mrps36	100	100
Q3THE2	Myosin regulatory light chain 12B	Myl12b	1.719	1.422
P52503	NADH dehydrogenase [ubiquinone] iron-sulfur protein 6. mitochondrial	Ndufs6	0.346	0.516
Q8BG18	N-terminal EF-hand calcium-binding protein 1	Necab1	2.643	2.811
Q61937	Nucleophosmin	Npm1	1.648	1.500
Q9Z0P4	Paralemmin-1	Palm	1.806	1.568
Q9QYX7	Protein piccolo	Pclo	1.652	1.487
Q6P8I4	PEST proteolytic signal-containing nuclear protein	Pcnp	1.599	1.497
Q80U04	E3 ubiquitin-protein ligase Praja-2	Pja2	100	100
Q9DBR7	Protein phosphatase 1 regulatory subunit 12A	Ppp1r12a	1.748	1.542
Q3UPH1	Protein PRRC1	Prrc1	2.161	0.010
E9PUL5	Proline-rich transmembrane protein 2	Prrt2	2.826	2.067
P32848	Parvalbumin alpha	Pvalb	0.376	0.527
P54728	UV excision repair protein RAD23 homolog B	Rad23b	1.416	1.376
Q8VE37	Regulator of chromosome condensation	Rcc1	0.010	0.010
O54916	RalBP1-associated Eps domain-containing protein 1	Reps1	1.731	1.454
P47915	60S ribosomal protein L29	Rpl29; Gm8210	5.849	3.075
P47955	60S acidic ribosomal protein P1	Rplp1	2.454	2.451
P99027	60S acidic ribosomal protein P2	Rplp2	2.593	2.418
P62849	40S ribosomal protein S24	Rps24	1.315	1.351
Q9ES97	Reticulon-3	Rtn3	1.578	1.329
Q9Z2G6	Protein sel-1 homolog 1	Sel1l	1.369	1.438
Q80Z38	SH3 and multiple ankyrin repeat domains protein 2	Shank2	2.126	1.742
Q80TR4	Slit homolog 1 protein	Slit1	0.476	0.441
O55042	Alpha-synuclein	Snca	1.959	1.649
Q91ZZ3	Beta-synuclein	Sncb	2.472	2.030
Q9CY18	Sorting nexin-7	Snx7	1.826	1.520
Q8BTI8	Serine/arginine repetitive matrix protein 2	Srrm2	1.772	1.482
Q08943	FACT complex subunit SSRP1	Ssrp1	0.674	0.625
P11031	Activated RNA polymerase II transcriptional coactivator p15	Sub1	1.550	1.368
F6SEU4	Ras/Rap GTPase-activating protein SynGAP	Syngap1	1.413	1.336
Q8CC35	Synaptopodin	Synpo	1.311	1.332
Q8R0A5	Transcription elongation factor A protein-like 3	Tceal3	7.815	4.891
Q8CCT4	Transcription elongation factor A protein-like 5	Tceal5	3.555	2.319
Q8R3L2	Transcription factor 25	Tcf25	100	100
Q64511	DNA topoisomerase 2-beta	Top2b	0.659	0.611
Q8BJU2	Tetraspanin-9	Tspan9	1.989	2.104
O70480	Vesicle-associated membrane protein 4	Vamp4	0.724	0.720
Q6PEV3	WAS/WASL-interacting protein family member 2	Wipf2	2.069	2.458
Q80TK0	AP2-interacting clathrin-endocytosis protein	Kiaa1107	2.231	1.873
Q0PMG2	MAM domain-containing glycosylphosphatidylinositol anchor protein 1	Mdga1	2.969	3.073

## Data Availability

The data presented in this study are available on request from the corresponding authors.

## Supplementary Materials

The following are available online at https://www.mdpi.com/article/10.3390/ijms22084290/s1, Supplementary dosimetric information, Figure S1: PC1 loading from PCA of Raman spectral data, Figure S2: Ponceau staining for the immunoblotting of the hippocampus samples 15 days after PBI or WBI, Figure S3: network analysis of the shared proteins between PBI 2.0 Gy and WBI 2.0 Gy, Figure S4: Ponceau staining for the immunoblotting of the hippocampus samples 6 months after PBI or WBI, Table S1: Hippocampal proteins in all treatment conditions, Table S2: Significantly deregulated proteins in hippocampus at 15 days after 0.1 Gy PBI, Table S3: Significantly deregulated proteins in hippocampus at 15 days after 0.1 Gy WBI, Table S4: Significantly deregulated proteins in hippocampus at 15 days after 2.0 Gy PBI, Table S5: Significantly deregulated proteins in hippocampus at 15 days after 2.0 Gy WBI, Table S6: Most significant biological processes in GO terms represented by the commonly deregulated proteins at 2.0 Gy PBI and 2.0 Gy WBI at 15 days, Table S7: All identified proteins in hippocampus after 6 months, Table S8: Significantly deregulated proteins in hippocampus at 6 months after 0.1 Gy PBI, Table S9: Significantly deregulated proteins in hippocampus at 6 months after 0.1 Gy WBI, Table S10: Significantly deregulated proteins in hippocampus at 6 months after 2.0 Gy PBI, Table S11: Significantly deregulated proteins in hippocampus at 6 months after 2.0 Gy WBI.

## Abbreviations

Cx43	Connexin43
DAG	Diacylglycerol
DCX	Doublecortin
DG	Dentate gyrus
FC	Fold-changes
GFAP	Glial fibrillary acidic protein
GJ	Gap junction
IP3	Inositol trisphosphate
NSC	Neural stem cells
PBI	Partial body irradiation/irradiated
PCA	Principal component analysis
RIBE	Radiation induced bystander effects
SGZ	Sub granular zone
SI	Sham irradiation/irradiated
Sox2	Sex determining region Y (SRY) box 2
TGF	Transforming growth factor
TNF	Tumor necrosis factor
WBI	Whole body irradiation/irradiated
