# Supplementary material for: Out-of-Field Hippocampus from Partial-Body Irradiated Mice Displays Changes in Multi-Omics Profile and Defects in Neurogenesis
Source: Int J Mol Sci. 2021 Apr 20;22(8):4290. doi: 10.3390/ijms22084290 (PMC8074756; doi:10.3390/ijms22084290)
Supplement: Supplementary file 1 [file ijms-22-04290-s001.zip › Supplementary dosimetric information.docx]

**Supplementary dosimetric information**: Dosimetry was carried out for mice irradiated at postnatal day 80 with 0.1, 0.25, and 2Gy of X-rays, with individual lead parallelepipeds used to protect the anterior two-thirds of the body, with the hindmost part directly exposed to radiation. Irradiation was delivered with a Gilardoni CHF 320 G X-ray generator operated at 60, 100, 250 kVp, 15 mA, with HVL = 1.6 mm Cu (additional filtration of 2.0 mm Al and 0.5 mm Cu). To insure conditions of extended phantom and full backscatter, irradiation was performed on a perspex rectangular solid phantom (length, width: 40 cm; height: 10 cm). At irradiation distance of 67.7 cm, the absorbed dose rate at the center of the irradiated volume in muscle was 1.03 Gy min-1 with relative expanded uncertainty of 10%, confidence level 95%.

Dose monitoring was made using a PTW 7862 large-size plane parallel transmission chamber connected to a PTW IQ4 electrometer. The absorbed dose delivered at a given depth in an extended muscle phantom was determined from the measured value of the air kerma on the basis of the “in-air method” (1), and using the function "Percentage Depth Dose" (2). Dose measurements were carried out by a cylindrical NE 2571 ionization chamber, coupled to a Farmer 2570/1 electrometer, calibrated in terms of Air Kerma at the Italian National Metrological Institute.

Experimental measurements were carried out to ensure that the out-of-field effects under study will not be the result of photons crossing the lead shield or deflected in the cap through the irradiated tissues. To verify this, the NE 2571 ionization chamber was set in the same position as the brain of the irradiated mice and inserted into the lead parallelepiped cap with the same characteristics of the shields used to partly protect the mice to be irradiated. This was repeated with or without a phantom. The estimation of dose into the shield resulting from the average of 10 measurements showed that, due to primary photons beneath the shields plus scattered radiation from X-ray deflection through irradiated tissues, for a 2.0 Gy dose at 250 kVp, there was a dose of 4 mGy to the shielded brain, corresponding to 0.2% of the total dose.
