# Supplementary figures and images for: Out-of-Field Hippocampus from Partial-Body Irradiated Mice Displays Changes in Multi-Omics Profile and Defects in Neurogenesis
Source: Int J Mol Sci. 2021 Apr 20;22(8):4290. doi: 10.3390/ijms22084290 (PMC8074756; doi:10.3390/ijms22084290)

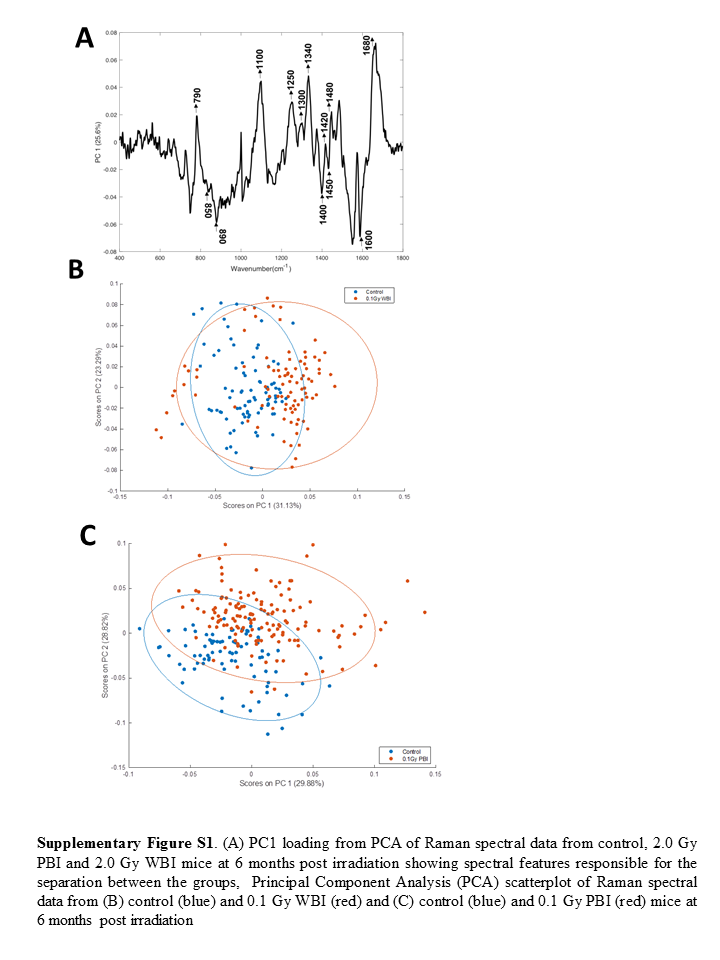

Supplement: Supplementary file 1 [file ijms-22-04290-s001.zip › Figure S1.tif]

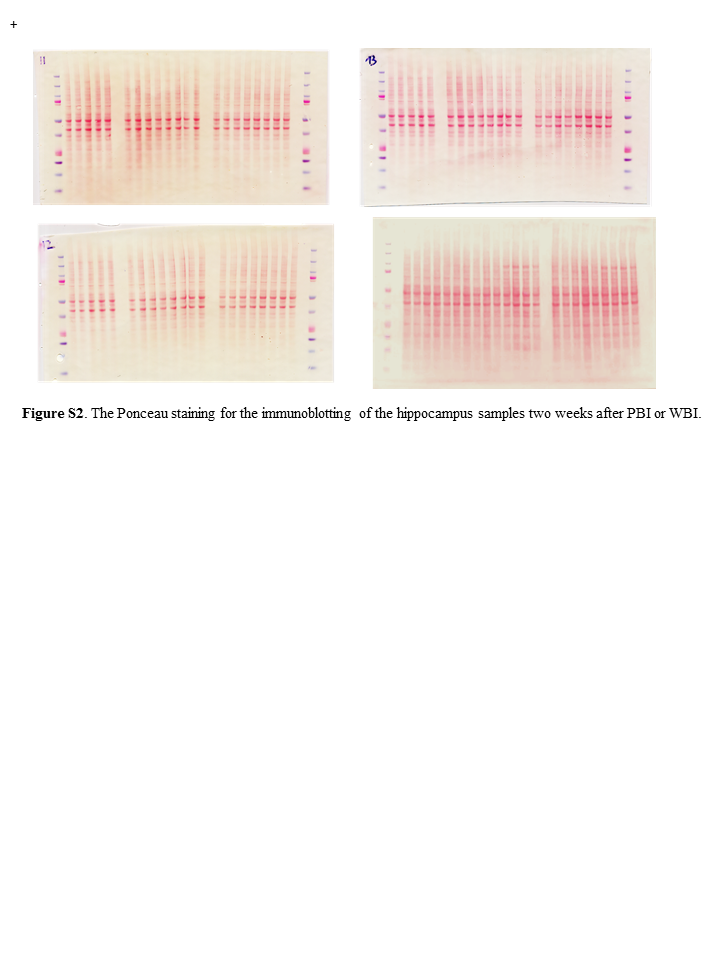

Supplement: Supplementary file 1 [file ijms-22-04290-s001.zip › Figure S2.tif]

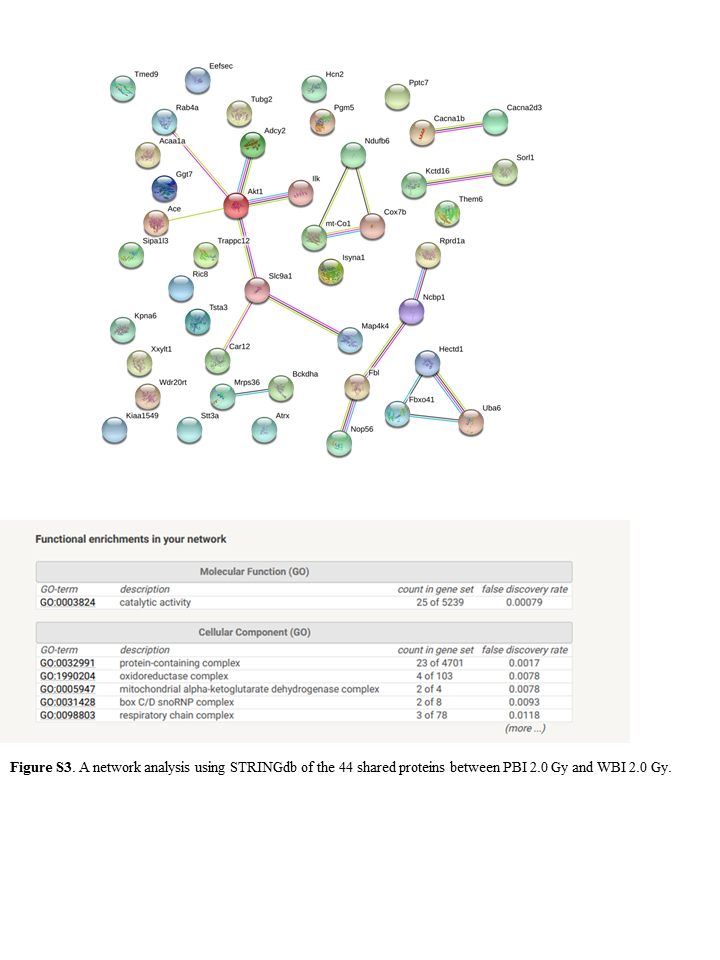

Supplement: Supplementary file 1 [file ijms-22-04290-s001.zip › Figure S3.tif]

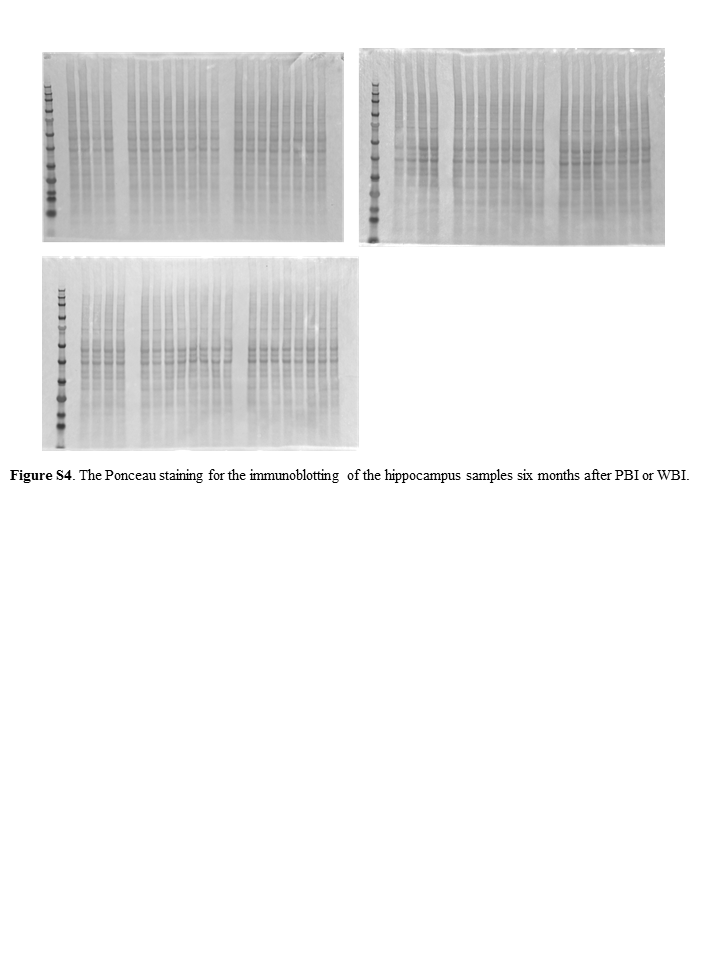

Supplement: Supplementary file 1 [file ijms-22-04290-s001.zip › Figure S4.tif]
